# Supplementary material for: Specific Detection of Influenza A and B Viruses by CRISPR-Cas12a-Based Assay
Source: Biosensors (Basel). 2021 Mar 19;11(3):88. doi: 10.3390/bios11030088 (PMC8003587; doi:10.3390/bios11030088)
Supplement: Supplementary file 1 [file biosensors-11-00088-s001.pdf]

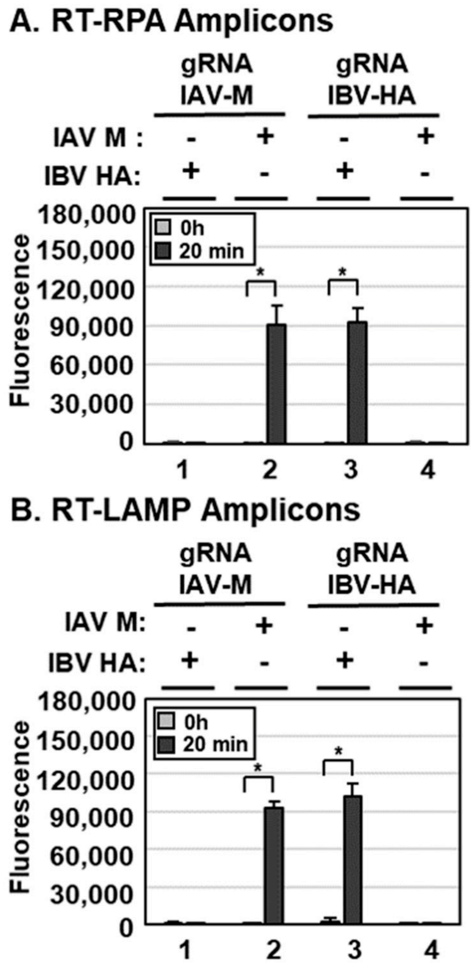


**Fig S1.** Specific detection of influenza virus types by the DETECTR assay. One hundred PFUs of IAV and IBV per reaction were used to amplify viral nucleic acids using either RT-RPA (A) or RT-LAMP (B) with primer sets specific for IAV M and IBV HA genes. DETECTR combined with fluorescence assays were performed with gRNAs targeting either IAV M or IBV HA genes to detect RT-RPA and RT-LAMP amplicons. Fluorescence signals of DETECTR on RT-RPA or RT-LAMP amplicons were saturated within 20 min. Values are presented as means ± s.d (error bars) (n = 3 replicates; $^{*}P < 0.05$ between samples, two-sample t-test).

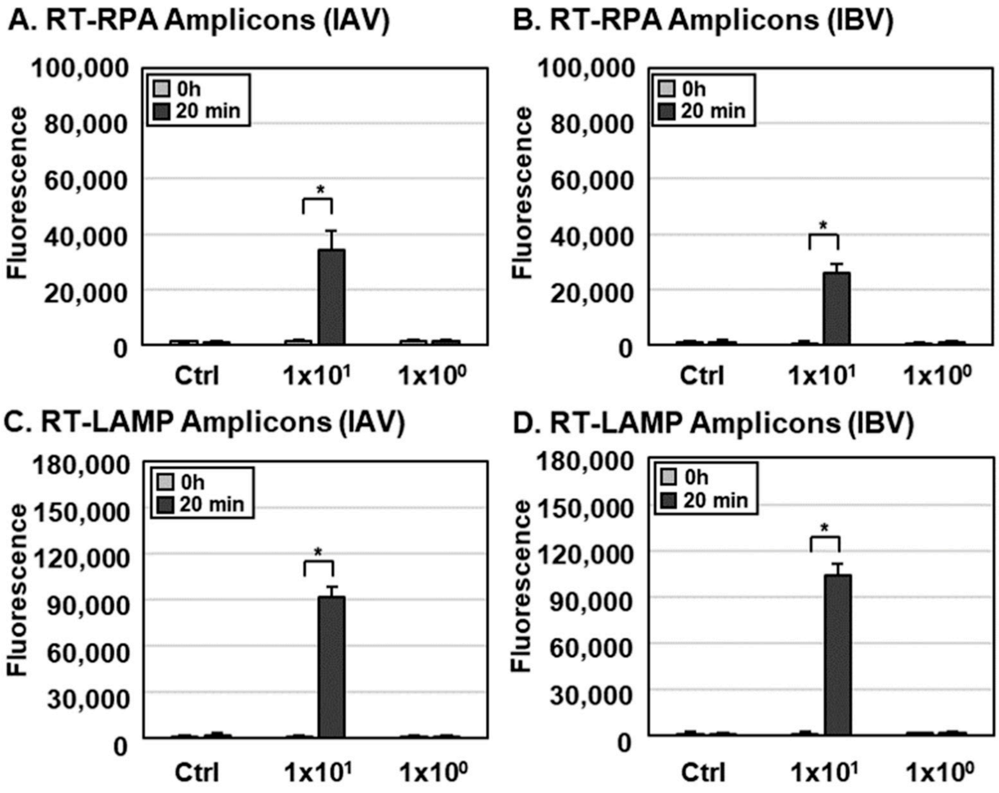


**Fig S2.** Sensitivity of the DETECTR assay for IAV and IBV in Saliva. (A-D) Different concentrations of IAVs (A and C) and IBVs (B and D) in human saliva (1.0 x $10^0$ to 1.0 x $10^1$ PFUs per reaction) were used to amplify viral nucleic acids using either RT-RPA (A and B) or RT-LAMP (C and D) with primer sets specific for the IAV M or IBV HA gene. RT-RPA and RT-LAMP amplicons were detected by DETECTR combined with fluorescence assay using gRNAs targeting the IBV HA gene. The fluorescence signals of DETECTR on RT-RPA or RT-LAMP amplicon were saturated within 20 min. Values are presented as means ± s.d (error bars) (n = 3 replicates; $^{*}P < 0.05$ between samples, two-sample t-test). C-line, control line; T-line, test line; Ctrl, control.
